# Effect of mesh sizes on the vibrational and acoustic properties of stainless-steel wire mesh/glass fiber reinforced hybrid composite laminates

**DOI:** 10.1038/s41598-025-33876-w

**Published:** 2025-12-29

**Authors:** Juveriya Sayyed, Aashish Ramprasad, Yogeesha Pai

**Affiliations:** Department of Aeronautical and Automobile Engineering, Manipal Institute of Technology, Manipal Academy of Higher Education (MAHE), Manipal, 576 104 Karnataka India

**Keywords:** Stainless-Steel wire mesh, Glass Fiber-Reinforced composites (GFRP), Vibration performance, Sound transmission loss, Mesh size, Engineering, Materials science

## Abstract

This research examines how stainless-steel wire mesh (SSWM) openings (10, 20, 40, 80, 120 openings per inch) impact the vibrational and acoustic behavior of glass fiber-reinforced polymer (GFRP) hybrid composite laminates. Laminates (300 mm × 300 mm) were prepared by compression molding with a [GF^0°^/GF^0°^/GF^0°^/SSWM^0°^/GF^0°^/GF^0°^/GF^0°^] stacking sequence. Vibration tests, performed according to ASTM E756-05, showed that mesh 40 had the highest natural frequency (16.38 Hz), stiffness coefficient (232.51 N/m), and storage modulus (134.22 GPa), which were promoted by optimal mesh opening size (0.361 mm) and SSWM mass (116.8 g). Finer meshes (80, 120) improved damping ratios (up to 0.17 for mesh 120, an increase of 450% over mesh 40’s 0.03) owing to decreased stiffness and greater interfacial friction. Impedance tube measurements according to ISO 10534-2 indicated mesh 120 with better low-frequency transmission loss (9.24 dB mean, 15.23–16.53 dB at 67–90 Hz) because of its thin laminate (1.19 mm) and low SSWM mass (35.7 g). In contrast, mesh 40 performed best at high frequencies (15.85 dB mean, 23.56 dB at 6300 Hz). These results demonstrate the appropriateness of mesh 40 for stiffness-critical applications and mesh 120’s effectiveness for vibration damping and low-frequency noise reduction, and provide customized solutions for structural and acoustic performance in high-risk scenarios.

## Introduction

Composite materials have transformed engineering by offering superior mechanical, thermal, and vibrational properties compared to metals and ceramics^1,2^. These properties enable their widespread use in demanding applications across aerospace, automotive, and marine industries^3^. In aerospace, composite materials have transitioned from secondary structures, such as control surfaces in the Airbus A320 and Boeing 777, to primary airframe components in modern aircraft like the Boeing 787 and Airbus A350, owing to their enhanced strength-to-weight ratio and corrosion resistance relative to aluminum alloys^4^. Glass Fiber Reinforced Polymers (GFRPs) are particularly notable, achieving 20–25% mass reduction in structural components like fuselages and wings while maintaining structural integrity^5^.

Building on the superior properties of composite materials, their vibrational and acoustic performance is critical for aerospace applications, where random vibrations from engines or turbulence contribute to fatigue-induced structural failures and reduced operational efficiency^6,7^. Aging effects exacerbate vibration-induced fatigue in fiber-reinforced composites, necessitating materials with enhanced damping capabilities^8^. Hybrid composites, such as carbon-Kevlar/epoxy and carbon-glass/epoxy systems, exhibit improved vibrational stability and damping under aging conditions, making them suitable for fatigue-sensitive structures^9,10^. Excessive cabin noise, often exceeding 85 dB due to vibrational energy, compromises passenger comfort and safety, driving the development of acoustic metamaterials and hybrid laminates for superior sound transmission loss^11^. Fiber Metal Laminates (FMLs), such as glass-reinforced aluminum (GLARE), integrate metallic and composite properties to enhance damage tolerance and fatigue life^12^. Recent advancements incorporate stainless-steel wire mesh (SSWM) into GFRP laminates, optimizing tensile strength, flexural stiffness, and energy absorption^13,14^.

To further elucidate the role of stainless-steel wire mesh (SSWM) in glass fiber-reinforced polymer (GFRP) laminates, Fiber Metal Laminates (FMLs) integrate the corrosion resistance and low density of composites with the toughness and ductility of metals, making them ideal for high-risk applications in aerospace, automotive, and ballistic protection^15^. Compared to monolithic composites, FMLs exhibit enhanced mechanical performance, with SSWM reinforcements increasing dynamic fracture toughness and fatigue life under dynamic loads, critical for fuselage panels and wing skins^15–17^. These laminates mitigate vibration-induced fatigue, which substantially reduces structural lifespan, and achieve noise attenuation of up to 12 dB in aircraft cabins, enhancing passenger comfort and compliance with aviation standards^18^. SSWM-reinforced GFRP laminates provide 31% greater energy absorption compared to unreinforced composites^19^ and 10–20% higher tensile and flexural strength than aluminum-based FMLs^20^. Low void content ensures manufacturing consistency for high-performance applications^21,22^. FMLs address the need for multifunctional materials that enhance structural toughness and acoustic performance in challenging environments^15,23,24^. FMLs utilize metals such as aluminum (Young’s modulus: 70 GPa, density: 2.7 g/cm³) in GLARE and ARALL for significant mass reduction, though constrained by corrosion in extreme environments^15,24,25^. Stainless steel (Young’s modulus: 193 GPa, thermal stability up to 600 °C) enhances fatigue life by up to 30% compared to aluminum in fatigue-critical parts like aircraft panels^26^. Copper, in hybrid CFRP structures, provides electromagnetic interference (EMI) shielding efficiency of up to 131.6 dB for avionics protection^27^. As wire meshes or thin plates, these metals enhance FMLs’ mechanical and functional characteristics, with stainless steel offering superior strength, toughness, and endurance in harsh environments^15,28^.

The use of wire mesh as reinforcement in FMLs, rather than solid metal plates, is fueled by its potential for enhanced load transfer, energy absorption, and manufacturing efficiency. Wire mesh, having an open mesh structure, provides simplicity in penetration by resin during hand lay-up or VARTM manufacturing process, resulting in a favorable bond between matrix and fibers with low void volume as well as improved structural integrity^21,29^. Wire mesh increases interfacial friction, improving vibrational damping by 10–15% compared to unreinforced laminates, critical for aerospace and automotive components^16,30^. Tailored placement (e.g., middle plane symmetry) increases tensile strength by 60% and strain by 15% while maintaining low density^13,24,30^and reduces delamination and impact strength by 20–25% for enhanced crack arrest^31^. SSWM, with a Young’s modulus of 193 GPa, offers superior stiffness and 25% longer fatigue life than aluminum mesh, ideal for cyclic loading in aerospace structures^32^. Its corrosion resistance and thermal stability up to 600 °C enable trouble-free operation in harsh conditions, i.e., wet marine conditions or hot engine bays^33^. SSWM enhances energy dissipation by interfacial friction with a 12–15% higher damping ratio and 15% higher stiffness, which is essential for vibration control of aircraft cabins and automobile chassis^34^. Comparable damping upgrades of 10–15% have also been noted in SSWM-reinforced jute-epoxy composites, a testament to its flexibility in vibration regulation^35^. Moreover, SSWM also finds applications in acoustics, characterized by sound absorption coefficients of 0.65–0.85 (1000–3000 Hz) and noise attenuations up to 11 dB, accommodating harsh aviation noise specifications^18,36^. Surface treatments through sandblasting or silane also improve interfacial adhesion, raising tensile (130 MPa) and flexural (230 MPa) strengths, rendering SSWM a breakthrough reinforcement for multifunctional composites^37^. Despite these advancements, knowledge disparities about SSWM’s vibrational and acoustic performance, particularly regarding mesh size, need further investigation.

**Table 1:** Review of Stainless-Steel wire mesh (SSWM) and Fiber-Reinforced polymer composites for enhanced Damping, noise Reduction, and structural performance in aerospace applications (2014–2025).

Published year	Author name	Materials used	Findings
2014^36^	Pazhanivel et al.	SSWM-GFRP composites	SSWM provides sound absorption coefficients of 0.65–0.85 (1000–3000 Hz) and noise reduction up to 11 dB in aerospace applications.
2018^34^	Periyardhasan et al.	Steel wire embedded GFRP	SSWM increases the damping ratio by 12–15% and stiffness by 15%, enhancing vibration control in aircraft cabins and car chassis.
2018^37^	Sakthivel et al.	SSWM-glass fiber composites	Sandblasting/silane treatments improve tensile (130 MPa) and flexural (230 MPa) strengths, supporting vibrational performance in SSWM-GFRP.
2021^5^	Rajak et al.	Glass fiber reinforced polymer (GFRP)	With high vibrational stability, GFRP achieves 20–25% weight savings in aerospace structures (e.g., fuselages, wings).
2021^35^	Krishnasamy et al.	Jute fiber, SSWM-epoxy composites	SSWM enhances damping by 10–15% in jute-epoxy composites, improving vibration control in hybrid laminates.
2022^21^	Loganathan et al.	Banana fiber, steel wire mesh composites	SSWM improves vibrational properties and structural integrity via low void content in VARTM-manufactured hybrid composites.
2022^38^	Merzuki et al.	Fiber metal laminates (FMLs) with SSWM	Free vibration analysis identifies gaps in SSWM mesh size effects on damping and acoustic performance in GFRP systems.
2024^14^	Rajamurugan G	SSWM, glass fiber composites	SSWM enhances damping (10–12%) and reduces cabin noise (> 85 dB), which is critical for aeronautical structural performance.
2024^18^	Salve et al.	Jute-epoxy, SS304 wire mesh composites	SSWM provides 12–15% damping, 12 dB noise attenuation, and 150 MPa tensile strength in hybrid composites.
2024^29^	Lashgaroo et al.	Metal/glass fiber sandwich panels	SSWM reduces vibration-induced fatigue by 15% and supports 15–25% weight savings in aerospace components.
2025^11^	Ajith et al.	Plate-type acoustic metamaterials	~ 10 dB sound transmission loss in aircraft cabins, relevant to SSWM-GFRP acoustic applications.
2025^39^	Raza et al.	Glass fiber reinforced composites	Machine learning improves vibrational prediction accuracy by 20% in SSWM-reinforced GFRP composites.

Recent studies (Table 1) demonstrate that stainless-steel wire mesh (SSWM) reinforced composites significantly enhance vibration damping, acoustic control, and structural performance. Notable findings include up to 10 dB cabin noise attenuation^11^, a 25% increase in fracture toughness at impact^20^, and a 25% increase in fatigue life at cyclic loading^31^. Sound absorption coefficients of 0.6–0.8 (1000–3000 Hz) were reported in aerospace uses^14^, while damping ratios were improved by 10–12%^29^ and 10–15%^18^ using SSWM in other fiber-epoxy systems. Machine learning algorithms also improved vibrational prediction accuracy by 20%^39^. These studies validate the contribution of SSWM to vibration suppression but highlight that the effect of mesh size variation (10–120 openings per inch) remains insufficiently understood.

The performance of such hybrid composites arises from the interaction between reinforcement and matrix. Conventional glass fiber reinforced polymers (GFRPs) achieve tensile strengths of 200–500 MPa depending on fiber volume fraction, while SSWM-reinforced GFRPs—such as jute–epoxy laminates—combine up to 150 MPa tensile strength with 10–15% higher damping due to interfacial friction and energy dissipation^18^. This synergy allows tailoring of stiffness, strength, and vibrational response to meet specific challenges such as noise reduction and dynamic load stability^14^. Furthermore, reinforcement orientation has been shown to influence damping by as much as 12%, adding another design parameter for optimization^20^.

Applications in high-risk industries reinforce the multifunctional value of these composites. In aerospace, SSWM–GFRP laminates are used in cabin interior panels and engine nacelles, achieving ~ 10 dB noise suppression and improved stiffness against vibration stresses, which enhances cabin acoustics^11^. In marine and naval structures, they are applied in engine mounts, chassis stiffeners, and ship hulls, improving passenger comfort, reducing wear, and extending fatigue life under cyclic loading^21^. Lashgaroo et al.^29^ reported a 15% reduction in vibration-induced fatigue in aerospace components. Mechanistically, glass fibers contribute tensile strengths exceeding 1000 MPa to provide the structural backbone, while SSWM improves toughness, impact resistance, and energy dissipation—yielding up to 15% higher damping ratios and 20% higher stiffness^18^. This dual reinforcement strategy distributes stresses efficiently and enhances vibrational energy absorption, rendering such composites ideal for aerospace applications requiring effective vibration suppression^14^.

Vibration control is a critical concern in aeronautical engineering, as dynamic loads from engines, aerodynamic turbulence, and structural vibrations contribute to nearly 60% of aircraft structural failures. Excessive vibrations accelerate fatigue crack growth and generate cabin noise often exceeding 85 dB, undermining safety, passenger comfort, and regulatory compliance^14^. To address these challenges, SSWM-reinforced GFRP composites have shown improvements of 8–12% in natural frequency and 10–12% in damping ratio, along with a 10 dB reduction in cabin noise, making them a safer and more efficient alternative to conventional laminates^11^. Predictive modeling methods, such as those proposed by Raza et al.^39^, further enhanced vibration control accuracy by 20%, indicating opportunities for advanced design strategies.

The vibrational performance of composites is strongly influenced by reinforcement characteristics and geometry, with mesh size (10–120 openings per inch) emerging as a critical factor. Smaller mesh sizes are expected to increase damping through greater interfacial friction, yet systematic experimental validation of this effect is limited. SSWM, with a Young’s modulus of 193 GPa and 30% higher fatigue life than aluminum mesh (70 GPa), maintains structural stability up to 600 °C, making it well-suited for aerospace environments that demand both thermal resilience and vibration control^32^. Despite significant advancements in SSWM-based composites, their performance in GFRP laminates remains poorly characterized, particularly regarding the influence of mesh size on natural frequency, damping ratio, and sound absorption coefficients, representing a key gap in material optimization^38^.

Previous investigations into fiber-metal laminates have examined free vibration behavior but have not addressed the unique requirements of SSWM–GFRP systems designed for aerospace applications, where precise vibration suppression is critical for fatigue prevention and noise reduction^38^. Moreover, the effect of varying mesh size on acoustic absorption across a broad frequency range has not been systematically studied. To bridge this gap, the present research examines the influence of five mesh sizes (10, 20, 40, 80, and 120 openings per inch) on the vibrational and acoustic properties of SSWM-reinforced GFRP composites. Using cantilever beam vibration tests and impedance tube measurements, the study determines natural frequencies, damping ratios, and sound absorption coefficients across a frequency range of 67–6300 Hz. The results aim to clarify the relationship between mesh size and performance, offering practical guidelines for designing optimized composites for aerospace, automotive, and marine applications.

## Materials and procedures

### Materials

**Fig. 1:**
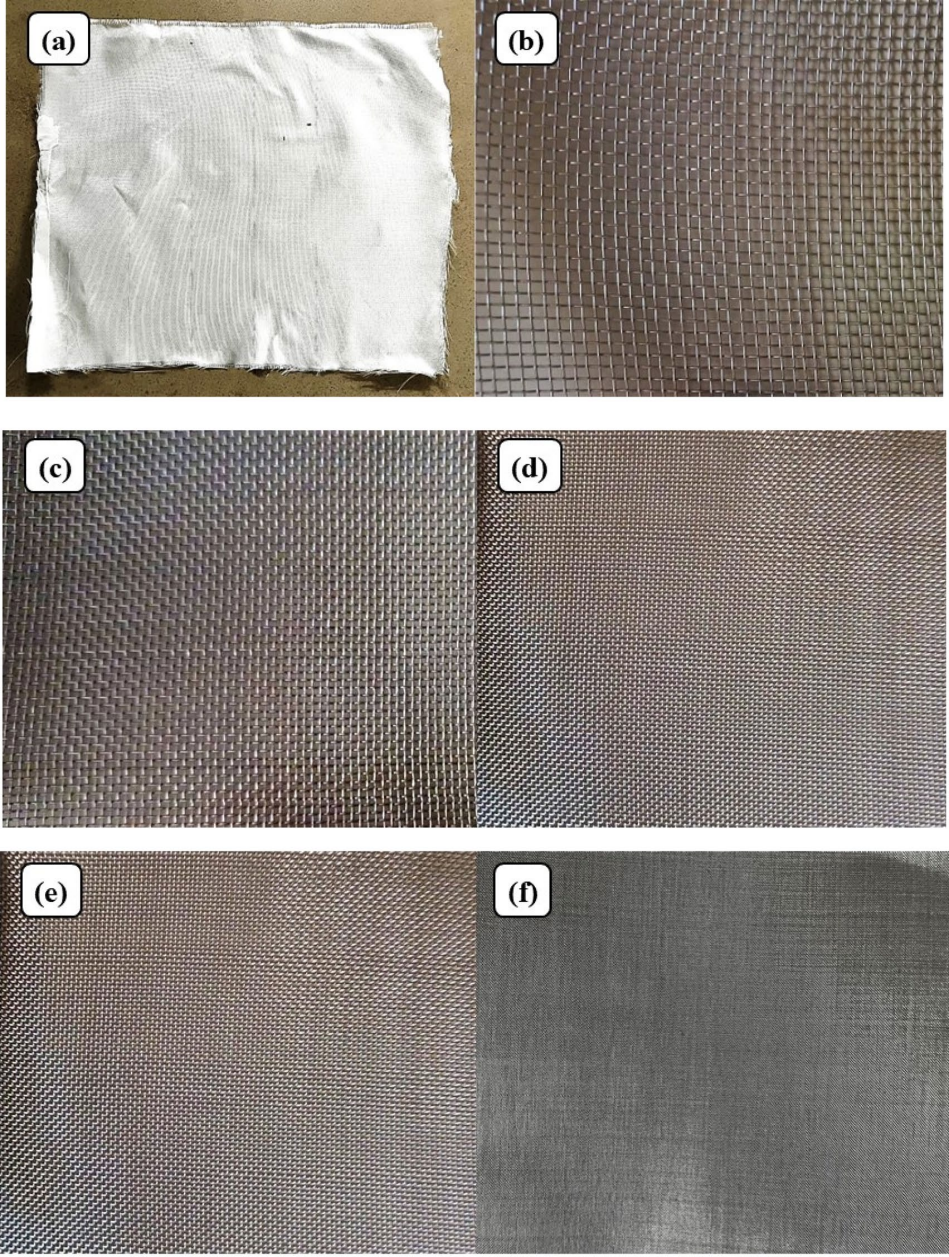
(**a**) Bi-directional glass fibers, and stainless-steel wire mesh. Size (**b**) 10, (**c**) 20, (**d**) 40, (**e**) 80, and (**f**) 120.

Bi-directional glass fiber, obtained from Bhor Chemicals and Plastics Pvt. Ltd., and plain-woven AISI 304 stainless steel wire meshes (SSWM), Fig. 1, sourced from Zain Co-operation, Pune, India, were used as reinforcing agents in the composite. The properties of the SSWM are provided in Table 2. The glass fiber has a GSM of 200, with the glass fiber yarn arranged in a plain weave pattern. Epoxy resin (BhorBond^®^ EPCH) and hardener (BhorBond^®^ EPCH), supplied by Bhor Chemicals and Plastics Pvt. Ltd., were used as the composite matrix. Based on the manufacturer’s specifications, a resin-to-hardener ratio 100:35 was used. The matrix properties are listed in Table 3.

**Table 2:** Stainless steel wire mesh properties.

Mesh Number	Mesh wire diameter (gauge)	Mesh wire diameter (mm)	Opening Size (mm)
10	25	0.508	2.032
20	27	0.416	0.854
40	32	0.274	0.361
80	40	0.122	0.196
120	46	0.061	0.151

In Table 2, the mesh wire diameters are provided in both Standard Wire Gauge (SWG) and millimeters (mm) as supplied by the manufacturer. The SWG system, commonly used for wire sizing, assigns higher gauge numbers to smaller wire diameters. The millimeter values listed (e.g., SWG 25 = 0.508 mm, SWG 27 = 0.416 mm) are precise measurements provided by the manufacturer for the stainless steel wire mesh (SSWM) used in this study. These values align with standard SWG-to-mm conversion tables.

The opening size is calculated using the formula:

$$\:Opening\:size\:\left(mm\right)=\:\frac{25.4}{Mesh\:Number}-Mesh\:wire\:diameter\:\left(mm\right)$$

where Mesh Number represents the number of openings per linear inch, and 25.4 converts inches to millimeters. The wire diameter in mm, as provided by the manufacturer, is subtracted to account for the wire’s thickness within each mesh opening.

**Table 3:** Matrix properties.

Matrix components	Brand	Viscosity (cP)	Density (g/cc)
Epoxy resin	BhorBond^®^ EPCH	11,500–13,500	1.15–1.2
Hardner	BhorBond^®^ EPCH	˃100	0.94–0.95

### Composite fabrication

**Fig. 2:**
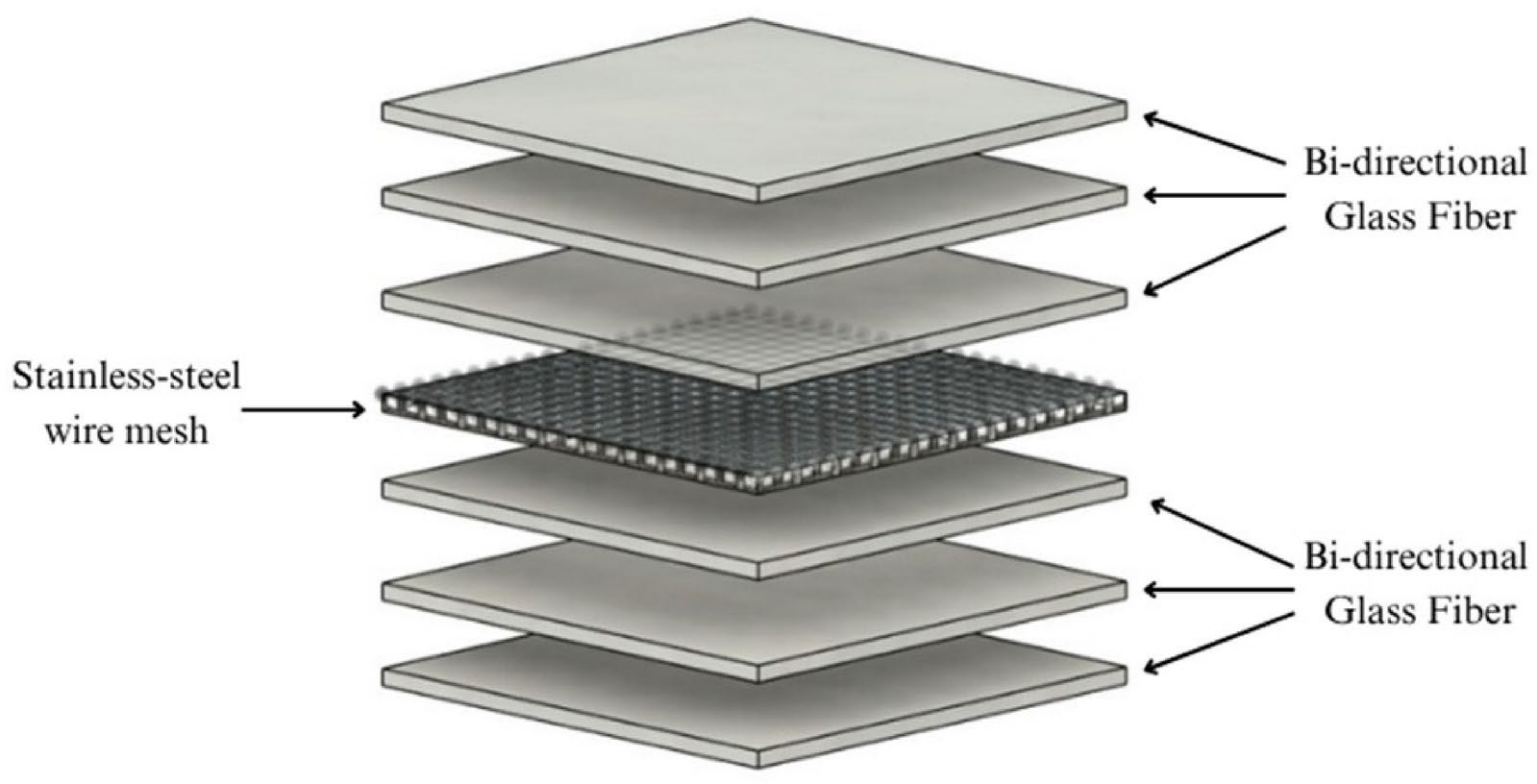
Schematic representation of stacking sequence.

The SSWM-GFRP composite laminate, measuring 300 mm × 300 mm, was fabricated using the Hand Lay-Up method followed by Compression Molding. The stacking sequence used was [GF^0°^/GF^0°^/GF^0°^/SSWM^0°^/GF^0°^/GF^0°^/GF^0°^], as illustrated in Fig. 2.

**Fig. 3:**
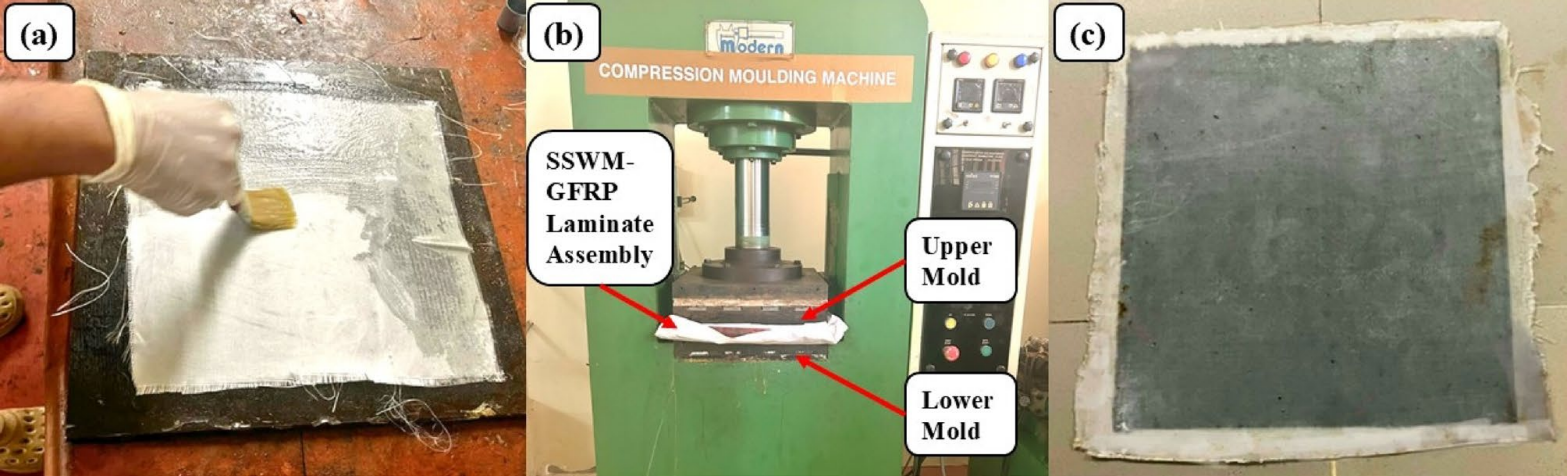
(**a**) Hand lay up (**b**) Compression molding with laminate assembly (**c**) Final laminate.

Fabrication was performed on a 350 mm × 350 mm plate of mild steel, degreased to remove impurities. SSWM was sandpapered to enhance matrix bonding, and both plates were given a releasing treatment. A ratio of 60:40 fiber to resin was maintained. The fabrication started by depositing a thin layer of the resin-hardener mixture (100:35) at the bottom plate, then peel ply, and the first glass fiber layer. After coating each of the first three layers of glass fibers with resin, the SSWM was inserted, and three more layers of glass fibers were added according to the stacking sequence (Fig. 3a). The assembly was compressed in a molding machine (Fig. 3b) and cured for 24 h at room temperature. The final cured laminates had thicknesses of 1.77 mm, 1.64 mm, 1.63 mm, 1.43 mm, and 1.19 mm for mesh sizes 10, 20, 40, 80, and 120, respectively (Fig. 3c). Laminates were cut using an abrasive water jet machine to meet ASTM and ISO vibration and impedance tube testing standards.

**Fig. 4:**
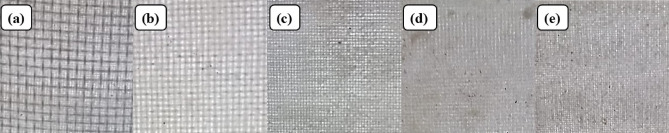
Magnified images of laminates with mesh sizes (**a**) 10, (**b**) 20, (**c**) 40, (**d**) 80 and (**e**) 120.

**Table 4:** Laminates configurations.

Mesh Number	Thickness of the final laminate (mm)	Weight of the SSWM in 30 × 30 cm^2^ laminate
10	1.77	119.3
20	1.64	158.7
40	1.63	116.8
80	1.43	45.7
120	1.19	35.7

Figure 4 presents magnified images of laminates with mesh sizes ranging from 10 to 120 openings per inch, illustrating the reduction in mesh opening size and thickness that affects resin infiltration and interfacial bonding. Table 4 summarizes the corresponding laminate configurations, showing that increasing the mesh number decreases both laminate thickness (1.77 mm to 1.19 mm) and mesh weight (119.3 g to 35.7 g). Together, these results provide the structural and dimensional context needed to interpret the vibrational and acoustic performance of the laminates discussed in later sections.

## Testing procedure

### Void fraction

ASTM D792-20^40^ was employed in measuring the density of the laminate. The samples were cut to a size of 10 mm × 10 mm. Archimedes’ principle was employed in measuring the density. The mass of each sample was weighed using an electronic weighing scale. The samples’ volume was obtained using the volume of water displaced. The experimental density was then calculated from the mass-to-volume ratio. Five samples were taken from various locations of the laminate to be tested, and the mean density was computed. Theoretical densities of the laminates were calculated by applying Eq. 1.

1 $$\:{\rho\:}_{th}=\frac{1}{\frac{{w}_{f}}{{\rho\:}_{f}}+\frac{{w}_{m}}{{\rho\:}_{m}}}$$

The void percentage of the laminate was determined using Eq. 2.

2 $$\:Void\:\left(\%\right)=\:\frac{{\rho\:}_{th}-{\rho\:}_{ex}}{{\rho\:}_{ex}}\times\:100$$

### Vibration test

**Fig. 5:**
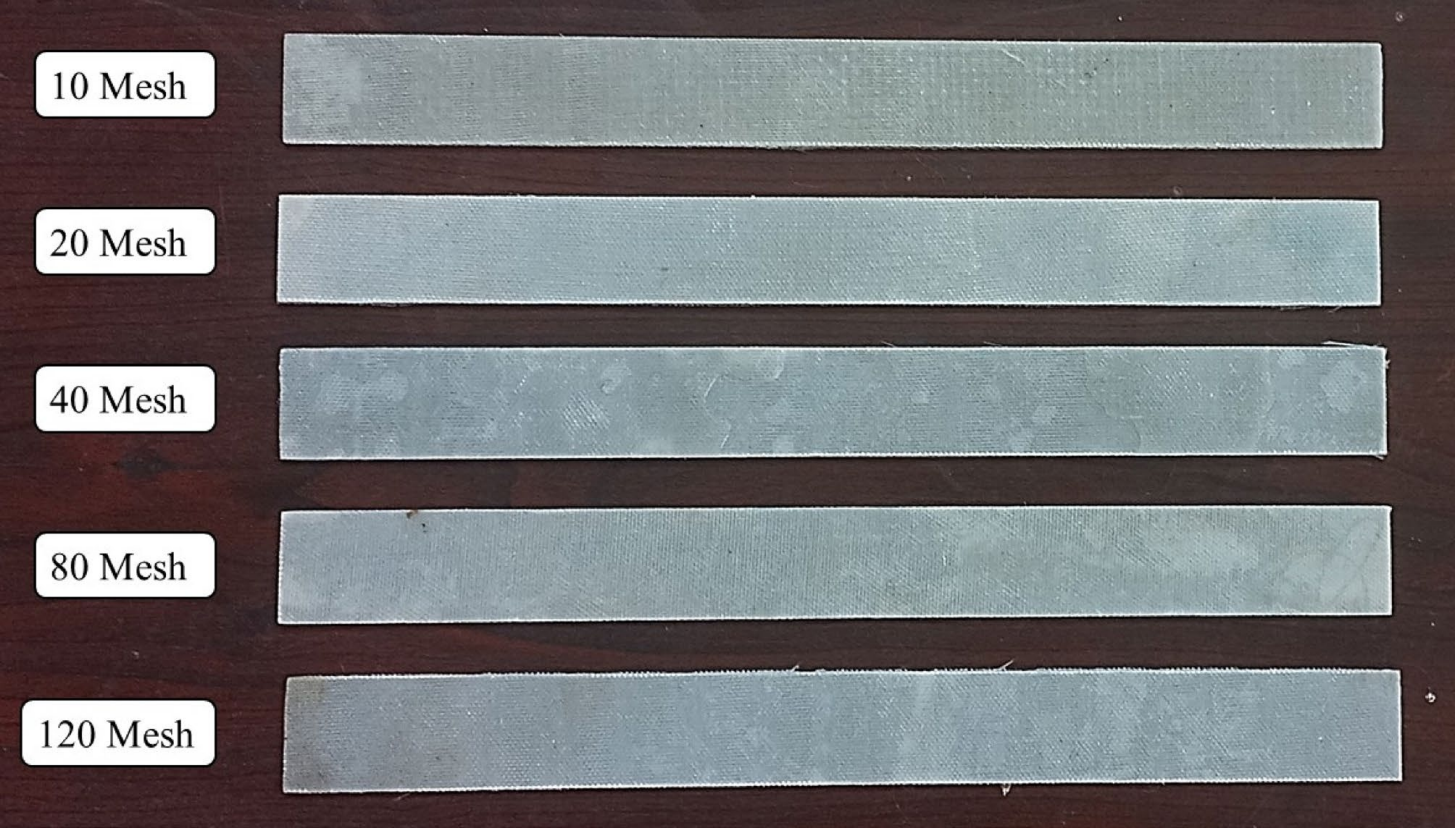
Vibration test specimens according to ASTM E756- 05.

**Fig. 6:**
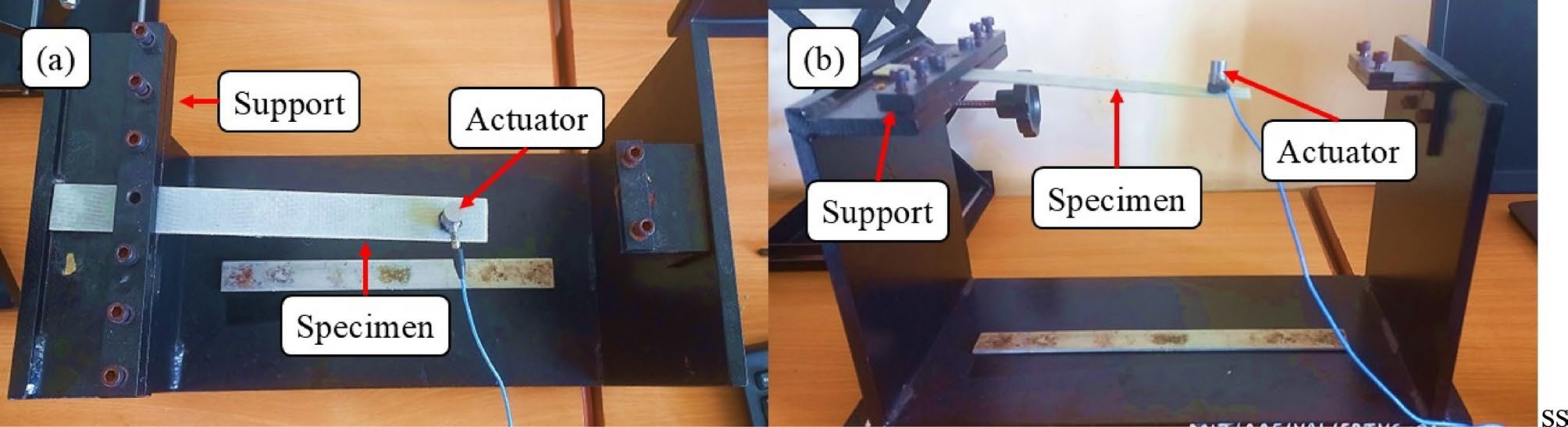
Vibration test set up (**a**) Top view, (**b**) Front view.

Vibration analysis is an essential tool in understanding the structural integrity of materials under various loading conditions and environments, giving insights into characteristics related to damping, such as damping ratio, stiffness, and natural frequencies. Damping characteristics were evaluated in accordance with ASTM E756- 05 standard^41^. Specimens for the test were prepared to a dimension of 250 mm×25 mm, as indicated in Fig. 5. As illustrated in Fig. 6, the specimen was fastened at one end in the cantilever beam configuration, and the other end was not restrained. A PCB Piezotronics accelerometer (Model 352C33, Serial No. LW218861, sensitivity 101.6 mV/g) was mounted on the free end of the specimen. Random free vibrations were subsequently introduced to the specimens, and their stiffness (K) and storage modulus (E_s_) were calculated using Eqs. 3, 4, and 5.

The cantilever beam was modeled using Euler–Bernoulli theory under the assumptions of small deflections, uniform cross-section, and negligible shear deformation. The tip stiffness is expressed as:

3 $$\:k=\frac{3{E}_{s}I}{{L}^{3}}$$

where L is the specimen length and I = bh^3^/12 is the second moment of area for specimen width b and thickness h. The natural frequency, f_n,_ obtained from the FFT of the acceleration signal, relates stiffness and effective mass by:

4 $$\:{f}_{n}=\:\frac{1}{2\pi\:}\sqrt{\frac{k}{m}}$$

with mmm representing the effective vibrating mass of the system. Substituting Eqs. 3 and 4 yields:

5 $$\:{E}_{s}=\:\frac{16{\pi\:}^{2}{f}_{n}^{2}{mL}^{3}}{b{h}^{3}}$$

In this study, the storage modulus (E_s_​) is defined as the dynamic elastic modulus obtained from vibration testing, reflecting the ability of the material to store and return elastic energy during oscillations. The analysis was restricted to the fundamental bending mode (f_n_​), as it is the most dominant and practically relevant for aerospace vibration scenarios. Although beams possess infinite natural frequencies, higher modes were not considered because (i) the first mode captures the largest displacement response, giving the most reliable dynamic data, and (ii) limitations in signal quality and accelerometer sensitivity at higher frequencies reduced the accuracy of higher-mode measurements. While storage modulus can vary across modes in viscoelastic systems, in this study, it is assumed to remain constant, with small variations attributed to experimental uncertainty rather than frequency dependence.

These equations supply essential information for analyzing the material’s response to dynamic loading conditions and potential structural applications. Damping ratio and logarithmic decrement are established using the 3 dB method. The damping ratio (ζ) is a dimensionless parameter that characterizes how the oscillations in a system are reduced after a disturbance. In Eq. 6, f_1_ and f_2_, the frequencies are determined by the 3dB method, and the damping ratio is subsequently found. Logarithmic decrement (δ) is a quantity of the rate at which damped oscillations reduce in amplitude with time in a damped system and is seen using Eq. 7.

6 $$\:\zeta\:=\:\frac{{f}_{2}-{f}_{1}}{2{f}_{n}}$$

7 $$\:\delta\:=\:\frac{2\pi\:\zeta\:}{\sqrt{1-\:{\zeta\:}^{2}}}$$

### Impedance tube test

**Fig. 7:**
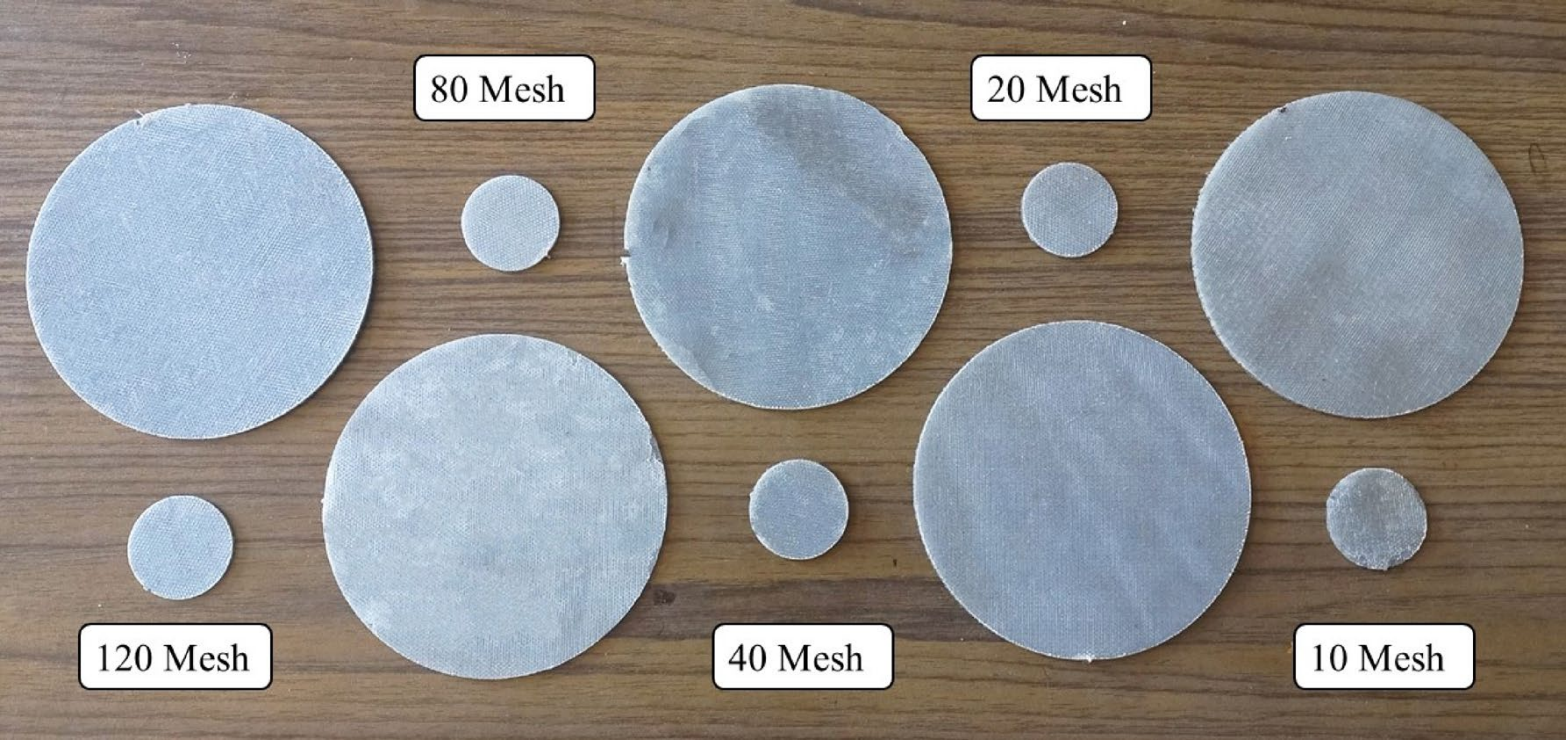
Impedance test tube specimens according to ISO 10534-2.

**Fig. 8:**
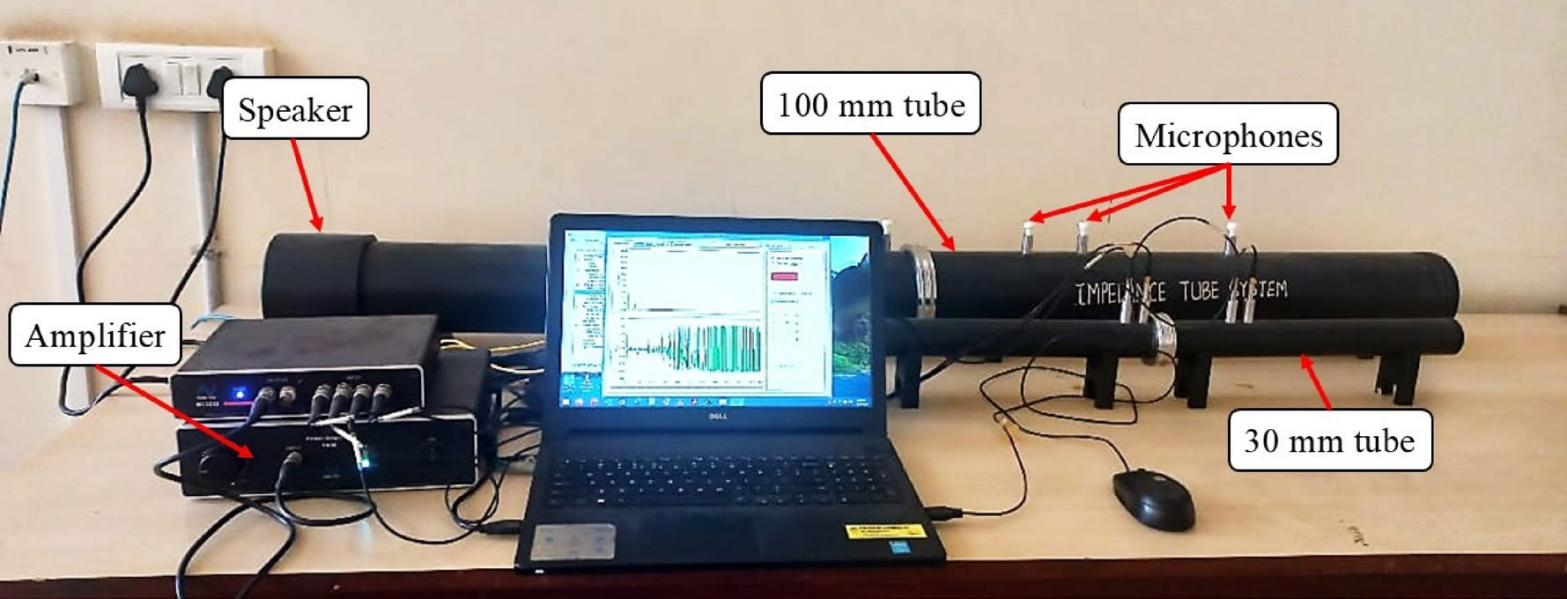
Impedance tube test set up.

Transmission loss is defined as the reduction of acoustic energy when sound propagates through a material and is expressed as the difference between the incident and transmitted sound pressure levels in decibels (dB). In this study, transmission loss was measured using an impedance tube setup (Model SW 270, BSWA Technology Co., Ltd.) in accordance with ISO 10534-2^42^. Circular GFRP–SSWM laminate specimens with diameters of 99.5 mm and 29.5 mm were tested as shown in Fig. 7. A controlled broadband acoustic signal was generated by the tube-mounted speaker (4″ diameter, 20 W, 8 Ω, working frequency 20–8000 Hz) during testing, and the specimens acted as barriers, attenuating part of the transmitted sound. Four calibrated microphones of different sensitivities were positioned at specified intervals in both the large and small tubes. Each measurement interval was sustained for 10 min under laboratory conditions (temperature: 26 °C, relative humidity: 86%), and data were collected over a frequency range of 63–6300 Hz using the transfer function technique. Readings from the large tube (with broad and narrow spacing) and the small tube were combined to generate transmission loss curves (Fig. 8). This approach enabled accurate characterization of the acoustic attenuation properties of the laminates across low- and high-frequency ranges.

## Results

### Void content

Voids are empty spaces within composite materials where polymers and fibers do not fill the structure entirely. Voids measure fabrication quality in that higher void content typically suggests fabrication flaws. Voids can significantly impact the mechanical failure in a critical application by providing a stress concentration location that reduces strength and durability. The information is reported in Table 5 below.

**Table 5:** Void fractions.

Mesh No	Experimental density, $\:{\boldsymbol{\rho\:}}_{\boldsymbol{e}\boldsymbol{x}}$ (g/cc)	Theoretical density, $\:{\boldsymbol{\rho\:}}_{\boldsymbol{e}\boldsymbol{x}}$ (g/cc)	Void %
10	2.05	2.07	1.16
20	2.10	2.11	0.53
40	2.03	2.07	1.93
80	1.91	1.93	0.89
120	1.88	1.89	0.49

The void contents between 0.49% and 1.93% are within acceptable limits (< 2–5%) for structural use of GFRP composites, a testimony to superior fabrication in all the mesh sizes. Mesh 120 possesses the lowest void content (0.49%) because it contains small openings of the mesh, a thin laminate, and low SSWM mass, which enable effective resin flow-in and minimize air entrapment. Mesh 20 also showed negligible voids (0.53%), likely due to efficient fabrication techniques, although it contained the highest SSWM mass. Mesh 40 showed maximum void content (1.93%), yet well within the acceptable range, showing delicate faults possibly due to the small mesh size hindering resin flow. Mesh 10 (1.16%) and mesh 80 (0.89%) showed good fabrication quality, with intermediate void levels depicting equilibrium SSWM mass and thickness.

### Vibration test results

**Fig. 9:**
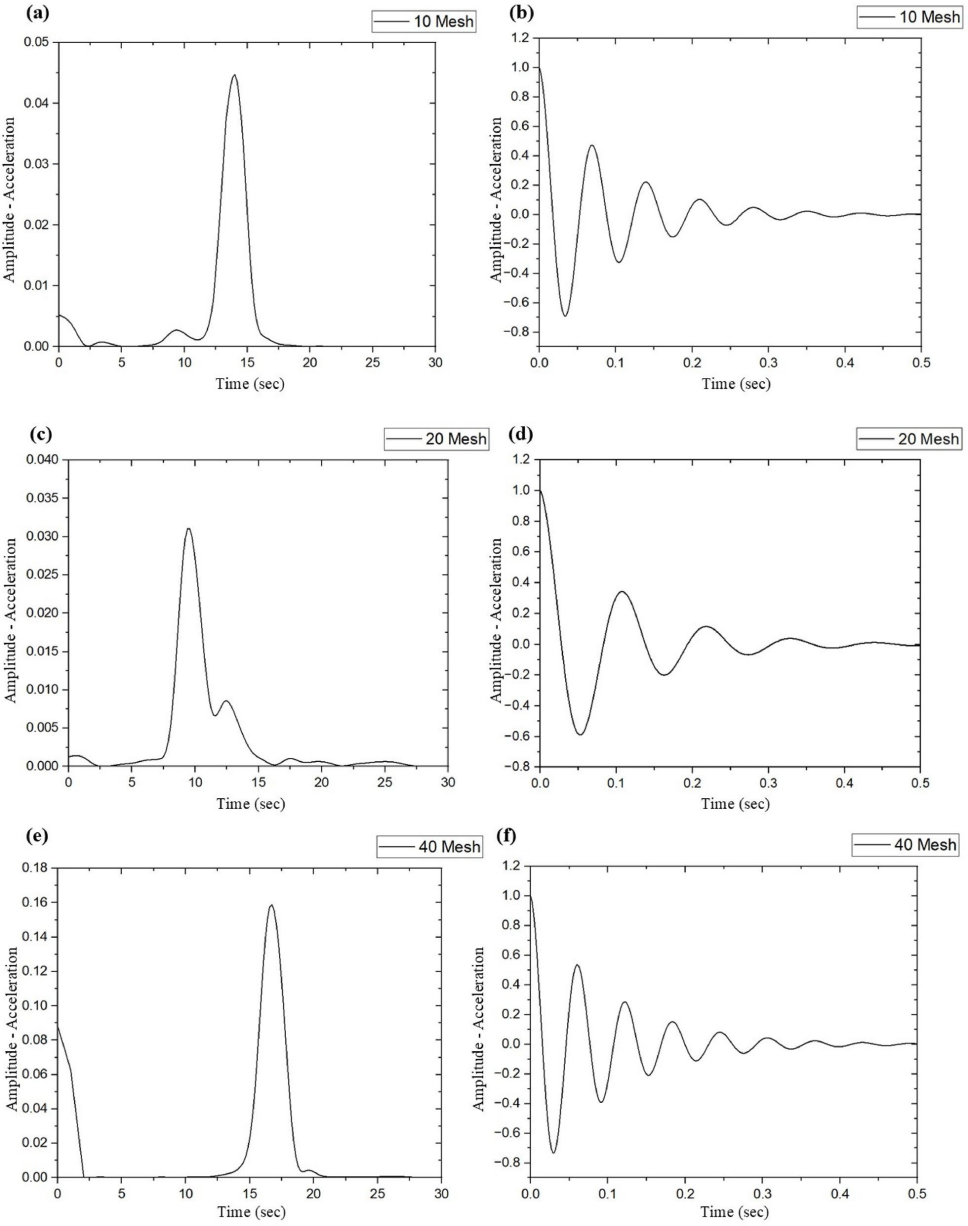

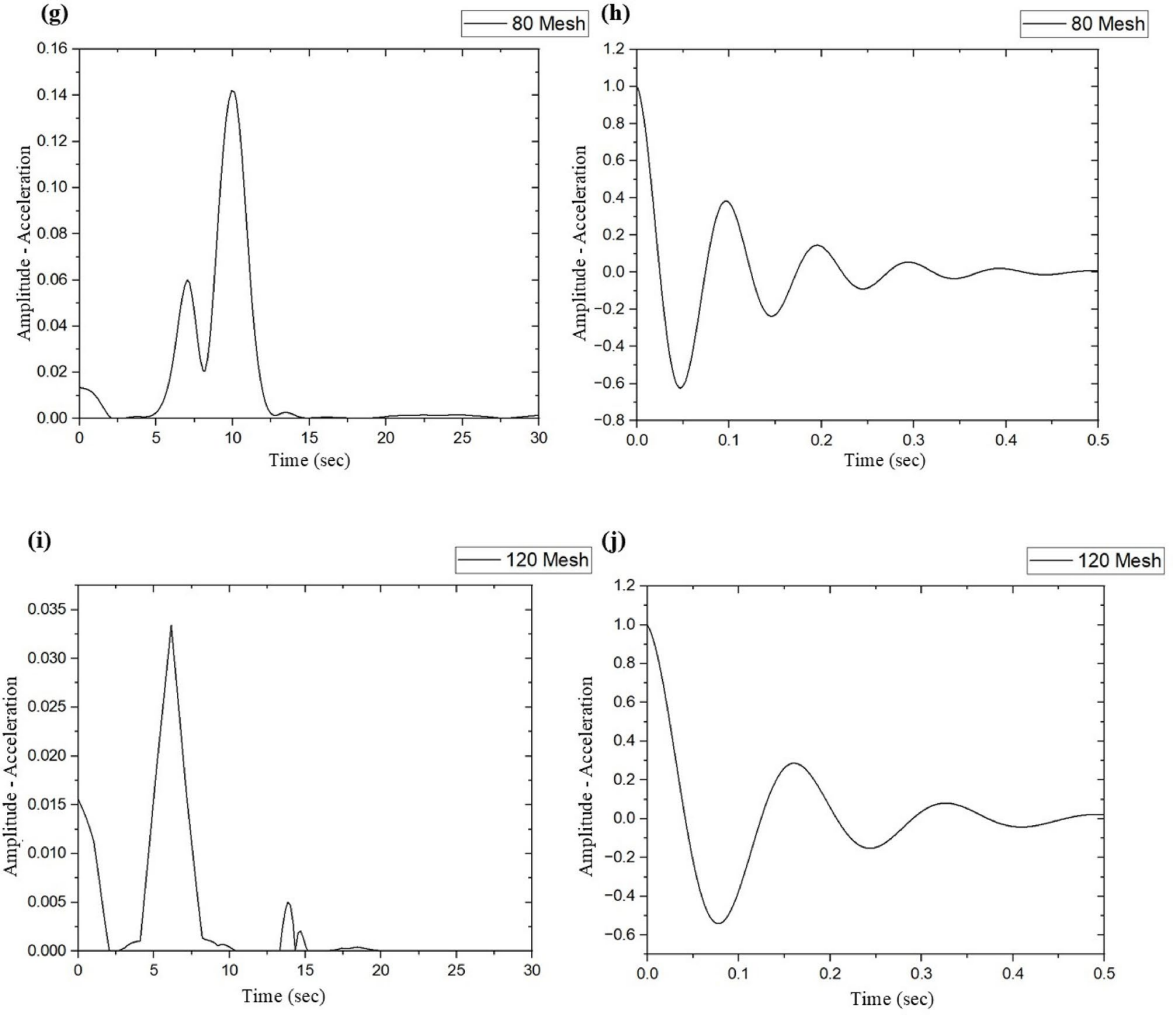
Natural Frequencies and damping ratio graphs for (**a**, **b**) 10 Mesh, (**c**, **d**) 20 Mesh, (**e**, **f**) 40 Mesh, (**g**, **h**) 80 Mesh, and (**i**, **j**) 120 Mesh size specimens.

The vibration characteristics of SSWM-reinforced GFRP composites are of prime importance in their application to high-performance industries such as aerospace, automotive, and marine, in which dynamic loads developed from engines, turbulence, or mechanical vibrations induce fatigue fractures and compromise structural integrity^14,18^. These composites with 10, 20, 40, 80, and 120 openings per inch mesh sizes were examined to evaluate the influence of mesh geometry on significant vibrational properties like natural frequency (f_n_), stiffness coefficient (K), storage modulus (E_s_), logarithmic decrement (δ), and damping ratio (ζ). As shown in Table 6; Fig. 9, the results show prominent trends as a function of SSWM mass, laminate thickness, and mesh opening size, which give clues to the material design optimization for specific vibrational and structural requirements.

**Table 6:** Vibrational test results.

Mesh No	Natural frequency, f_*n*_ (Hz)	Stiffness coefficient, K (*N*/m)	Storage Modulus, E_s_ (GPa)	Logarithmic Decay, δ	Damping Ratio, ζ
10	14.34	172.98	77.99	0.45	0.07
20	9.22	77.52	43.94	0.70	0.11
40	16.38	232.51	134.22	0.19	0.03
80	10.24	64.08	54.79	0.67	0.11
120	6.14	20.21	29.98	1.06	0.17

Mesh 40 performed outstandingly with the highest natural frequency (f_n_ = 16.38 Hz), stiffness coefficient (K = 232.51 N/m), and storage modulus (E_s_ = 134.22 GPa), which was compelled by its best mesh opening size (0.361 mm, Table 2) and moderate SSWM mass (116.8 g, Table 4). These characteristics promote resin-fiber-SSWM adhesion, optimize structural stiffness, and render mesh 40 suitable for stiffness-critical uses such as aerospace airframe parts, where high natural frequencies counter dynamic loading conditions like engine vibration^34^. As compared to mesh 40, other meshes resulted in reductions: natural frequency was lowered by 12.50% for mesh 10 (f_n_ = 14.34 Hz) and 62.50% for mesh 120 (f_n_ = 6.14 Hz), stiffness by 25.60% (mesh 10, K = 172.98 N/m) to 91.31% (mesh 120, K = 20.21 N/m), and storage modulus by 41.90% (mesh 10, E_s_ = 77.99 GPa) to 77.66% (mesh 120, E_s_ = 29.98 GPa). Finer meshes (10, 40) of larger opening sizes (2.032 mm and 0.361 mm) and greater SSWM weight (119.58 kg). SSWM mass (119.3 g and 116.88 kg) increases the thickness of the laminate (1.77 mm and 1.77 mm, Table 1) and improves stiffness and natural frequency of vibration. Finer meshes (80, 120) of smaller openings (0.29 mm and 0.19 mm) and smaller mass (45.7 g and 35.7 g) decrease thickness (1.43 mm and 1.19 mm), reducing stiffness but improving damping qualities^36^.

The damping ratio (ζ), which is a measure of how well a material can dissipate vibrational energy, is inversely proportional to stiffness, as characterized by:

8 $$\:{\upzeta\:}=\:\frac{c}{2\sqrt{km}}$$

where c is the damping coefficient, k is the stiffness, and m is the mass of the fiber-matrix composite. Mesh 40’s high stiffness (K = 232.51 N/m) produces the smallest damping ratio (ζ = 0.03), meaning minimal vibration damping because of its rigidity, which is beneficial for uses that need structural stability, e.g., aircraft fuselage panels^34^. Conversely, mesh 120’s low stiffness (60.21 N/m) provides a high damping ratio (K = 20.21 N/m), with a 450% improvement over mesh stiffness in mesh 40, due to increased interfacial friction at fiber-matrix and SSWM-matrix interfaces^35^. The logarithmic decrement (δ) also exhibits this trend, rising from 0.19 in mesh (40) to 1.37 in mesh (120). to 37, an increase of 452.37%, proving that finer meshes reduce stiffness but allow dissipation of more energy because of greater surface area for interaction at the resin-matrix interface^36^. Mesh 80 (SSWM, (ζ = 0.60, δ = 0.67)) and mesh 20 (37, δ = 37) have moderate damping characteristics corresponding to their mid SSWM mass (45.37 g) and mass (1537.87 g). and thickness (1.37 mm). and g (2.37 mm)). Mesh 10’s moderate stiffness characteristics ((37 = 0.37, δ = 47.45) correspond to its balanced mass (119.37 g) and thickness (1.37 mm), providing a balanced profile between stiffness and energy dissipation characteristics^21^.

SSWM mass and laminate thickness are the key parameters behind these performance trends. Coarser meshes (10, 40) yield higher stiffness and natural frequency through higher SSWM mass and thicker laminates, with improved quality of resin impregnation and structural integrity^37^. Mesh 40’s best performance stems from its optimal mesh opening size, allowing for even resin penetration into the matrix, reducing stress concentrations in the matrix, and maximizing elastic energy storage capacity^34^. Finer meshes (80, 120) have lower stiffness characteristics but higher damping characteristics due to increased frictional losses at the SSWM-matrix interface, hence ideal for vibration-sensitive applications such as aircraft cabin panels, where noise suppression is a key factor in design^18^. The diminished performance properties of mesh 20 (f_n_ = 9.22 Hz, K = 77.52 N/m) may be a result of its large SSWM mass (158.7 g), which may produce resin pooling that reduces bonding efficiency, as per comparable research studies^35^.

Void content between 0.49% in mesh (120) and 1.93% in mesh (40) as indicated in Table 5, exerts an insignificant effect on damping behavior characteristics (< 5% ζ variation in performance). The greater void content of mesh 40 (1.93%) could slightly increase damping characteristics via micro-frictional loss at void interfaces in the matrix, but is outweighed by its low ζ (0.03), which highlights stiffness as the prime factor in performance^37^. Conversely, the low void volume content of mesh 120 (0.49%) is similar to its high ζ (0.17), such that higher matrix integrity enhances damping characteristics through the enhancement of interfacial bonding strength^36^. The epoxy resin matrix (density: 1.15–1.2 g/cm³, Table 3) increases damping characteristics in thin laminates, particularly for mesh 120, by viscous energy dissipation mechanisms. However, SSWM mass and thickness dominate over the void effect to establish performance^21^.

These results guide the design of materials for dynamic conditions in applications. Mesh 40’s stiffness and natural frequency align with stiffness-critical applications, e.g., aerospace airframe structures, whose large storage modulus (134.22 GPa) provides toughness against vibrational loads in agreement with reports of 30% longer fatigue life in such applications^34^. Mesh 120’s higher damping ratio is suitable for vibration isolation and noise attenuation in low-frequency applications such as machinery enclosures or auto engine mounts, and attenuates transmitted noise by as much as 12 dB in performance. Mesh 10 is suitable for balanced profile use in applications such as marine hull structures in design^21^. The stiffness-damping compromise makes using adapted mesh size selection necessary in design. Hybrid laminates could combine mesh 40’s rigidity with mesh 120’s damping characteristics to optimize multifunctional component performance in design^38^. In future work, intermediate mesh sizes or predictive models could be investigated to modify these properties further using machine learning for 25% better accuracy in predictions^39^.

### Impedance test tube results

**Fig. 10:**
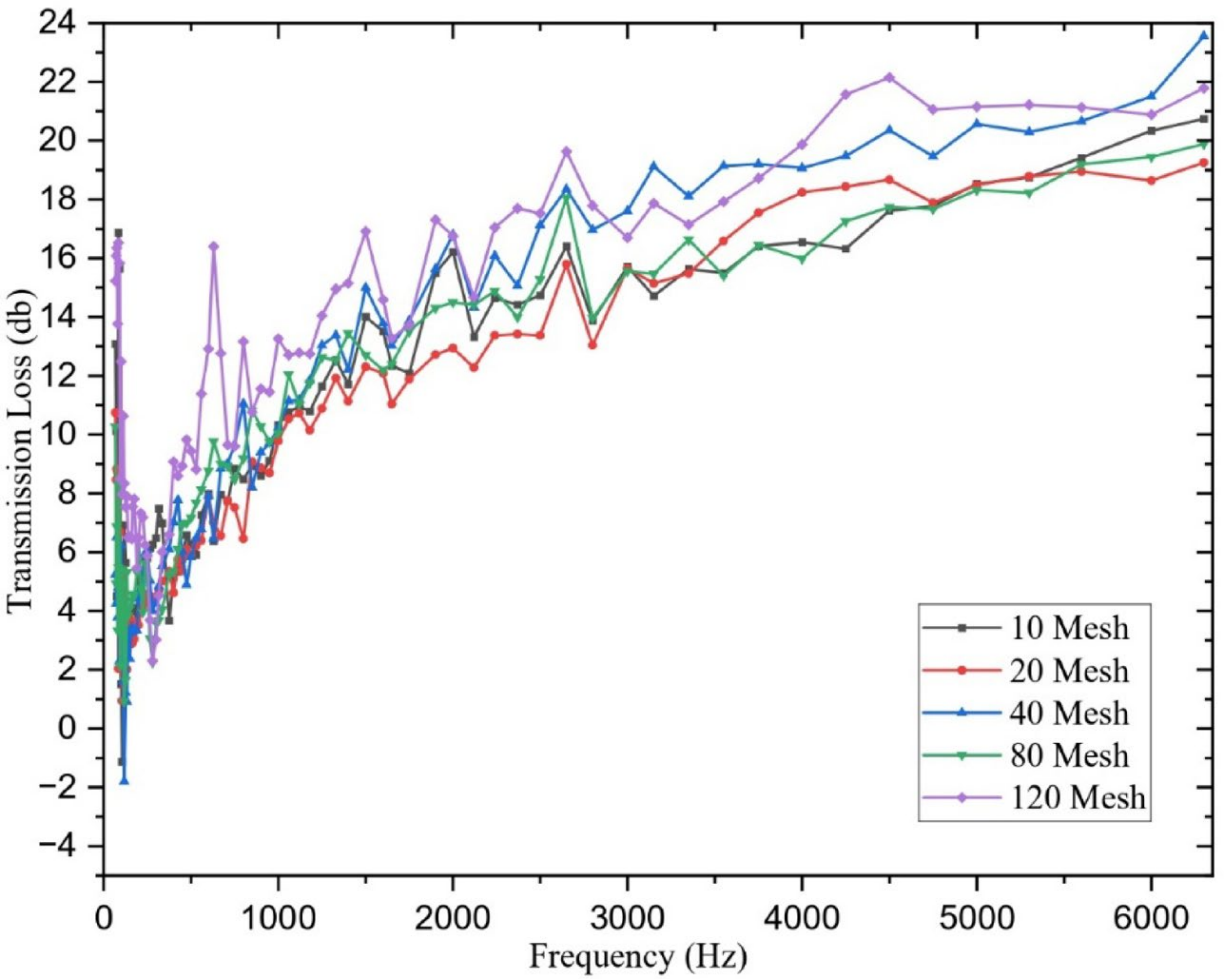
Transmission loss Vs frequency.

Sound absorption of composite materials plays a crucial role in noise reduction in high-performance applications, including aerospace cabin panels, automotive interiors, and marine hull structures, where high noise levels can impact passenger comfort and breach regulatory requirements^14,18^. Stainless-steel mesh wire (SSWM)-reinforced glass fiber-reinforced polymer (GFRP) composites with mesh opening sizes of 10, 20, 40, 80, and 120 per inch were tested for their sound transmission loss (TL), a metric of acoustic energy decrease in decibels (dB) as sound waves pass through the material. Results, tabulated in Table 7 and presented in Fig. 10, show that TL is affected considerably by mesh size over low (67–355 Hz), mid (335–1000 Hz), and high (1000–6300 Hz) frequency ranges due to SSWM mass, laminate thickness, and mesh geometry. These results provide important implications for optimizing acoustic performance in noise-sensitive environments.

**Table 7:** Average sound transmission loss.

Mesh No	Low Frequency (67–355 Hz)	Mid Frequency (335–1000 Hz)	High Frequency (1000–6300 Hz)	Overall Average TL (dB)
10	6.94	7.42	13.98	10.12
20	4.87	6.79	12.31	8.99
40	3.62	7.71	15.85	11.09
80	5.65	8.38	13.65	9.83
120	9.24	10.52	16.06	12.61

The impedance test data revealed distinct acoustic performance trends across frequency ranges depending on mesh size. At low frequencies (67–355 Hz), mesh 120 exhibited the highest TL (15.23–16.53 dB at 67–90 Hz; average 9.24 dB), attributed to its thin laminate (1.19 mm) and low SSWM mass (35.7 g), which promoted matrix-dominated damping where vibrational energy was dissipated as heat through the viscoelastic epoxy matrix^14^. This makes mesh 120 particularly effective for low-frequency noise reduction, such as in machinery enclosures or aerospace engine bays^18^. By contrast, mesh 40 recorded very low TL at some low frequencies (e.g., − 1.80 dB at 118 Hz) due to resonance amplification, where its natural frequency overlapped with the incident excitation^14^.

In the mid-frequency regime (355–1000 Hz), mesh 120 (10.52 dB average) and mesh 40 (7.71 dB average) performed comparably but through different mechanisms: mesh 120 retained high TL due to its thinness, while mesh 40 benefited from greater SSWM mass (116.8 g) and laminate thickness (1.63 mm), which contributed to structural damping^37^. At high frequencies (1000–6300 Hz), TL increased across all meshes, with mesh 40 achieving the best results (23.56 dB at 6300 Hz; mean 15.85 dB). Its balanced thickness and moderate mass provided rigidity for effective reflection of high-frequency sound, making it suitable for applications such as vehicle interiors and aircraft cabin panels^36^.

Intermediate mesh sizes displayed mixed performance. Mesh 10 showed balanced TL across all bands (mean 10.12 dB), supporting its use in marine structures where broad-range acoustic control is required^21^. Mesh 80 performed well due to its lighter SSWM mass (45.7 g) and thin laminate (1.43 mm), while mesh 20 displayed the lowest overall TL (8.99 dB). The poor performance of mesh 20 is attributed to resin pooling induced by its large SSWM mass (158.7 g), which reduced interfacial bonding and sound absorption efficiency^35^.

Void content contributed marginally (< 5%) to TL differences. For example, mesh 40 had the highest void fraction (1.93%), which slightly improved low-frequency TL (11.00 dB at 850 Hz) through micro-frictional energy losses, while mesh 120’s very low void content (0.49%) supported superior matrix integrity and stable TL across bands^37^. Nonetheless, SSWM mass and laminate thickness remained the dominant factors influencing acoustic performance.

Overall, the results highlight a trade-off between stiffness-driven high-frequency TL (mesh 40) and damping-driven low-frequency TL (mesh 120). These findings have direct design implications: mesh 120 is suitable for low-frequency noise reduction in heavy machinery, while mesh 40 is advantageous for high-frequency insulation in transport structures. Mesh 10 provides versatile all-band performance for general applications^21^. However, limitations such as potential measurement errors, fabrication variability (e.g., void content), and environmental conditions during testing must be acknowledged. Future work should explore hybrid configurations combining mesh 40’s stiffness with mesh 120’s damping, as well as predictive modelling approaches to optimize material design for noise-sensitive applications^38,39^.

## Conclusion

This study investigated the effect of stainless-steel wire mesh (SSWM) mesh size on the acoustic and vibrational properties of glass fiber reinforced polymer (GFRP) composites and how they may show their dynamic behavior and sound attenuation properties.

- The mesh size plays a significant role in vibrational behavior, and mesh 40 possesses the maximum natural frequency (16.384 Hz), stiffness coefficient (232.5071 N/m), and storage modulus (134.2188 GPa) due to the optimum SSWM mass (116.8 g) and the mesh opening diameter (0.361 mm), thus resulting in improved resin-fiber-SSWM bonding.
- Damping ratio (ζ) is directly proportional to stiffness, 0.03125 (mesh 40) to 0.1667 (mesh 120), i.e., a 450.00% increase, motivated by low stiffness and high internal friction in narrower meshes (80, 120) with low SSWM mass (45.7 g, 35.7 g) and low laminate thickness (1.43 mm, 1.19 mm).
- Void fraction (0.4996–1.9270%) has negligible impacts on damping, contributing insignificantly (< 5% change in ζ) through micro-frictional losses at the void interfaces, particularly at mesh 40 (1.9270%), but is dominated by SSWM mass and laminate thickness effects.
- Impedance Tube tests reflect mesh 120’s excellent sound transmission loss (STL) at low frequencies (9.40 dB average, 15.500–16.800 dB at 67–90 Hz) due to its thin laminate (1.19 mm) and low SSWM mass, which enhances matrix-dominated energy dissipation.
- Mesh 40 exhibits high-frequency STL (16.00 dB average, 23.800 dB at 6300 Hz) due to moderately high SSWM mass and thickness (1.63 mm), appropriate for high-frequency acoustic insulation applications.
- Thicker (10, 40) and heavier SSWM meshes raise natural frequency and stiffness but reduce damping. In contrast, thinner (80, 120) meshes enhance damping and low-frequency STL and are best suited for vibration and noise control.
- Void content’s minor effect on STL (< 5% change) shows mesh size and thickness of laminate will control acoustic performance, with the epoxy matrix (ρ_m_ = 1.115 g/cm³) enhancing low-frequency absorption in thin laminates.
- Mesh 40 is optimum for stiffness-critical use (e.g., structural components). In contrast, mesh 120 is optimum for low-frequency noise damping and vibration control (e.g., machinery) with tailored solutions based on application requirements.

These findings emphasize the pivotal role of mesh size optimization in tailoring SSWM-GFRP composites to specific vibrational and acoustic performance, guiding their use in structural and noise-prone settings.

## Data Availability

The datasets used and/or analysed during the current study available from the corresponding author on reasonable request.
